# Burosumab treatment of X-linked hypophosphatemia patients: interim analysis of the SUNFLOWER longitudinal, observational cohort study

**DOI:** 10.1093/jbmrpl/ziae079

**Published:** 2024-06-10

**Authors:** Toshimi Michigami, Hee Gyung Kang, Noriyuki Namba, Nobuaki Ito, Takuo Kubota, Ayumi Shintani, Daijiro Kabata, Masanori Kanematsu, Yayoi Nishida, Seiji Fukumoto, Keiichi Ozono

**Affiliations:** Department of Bone and Mineral Research, Osaka Women’s and Children’s Hospital, Osaka Prefectural Hospital Organization, Osaka 594-1101, Japan; Department of Pediatric Nephrology, Seoul National University Children’s Hospital, Seoul 03080, Republic of Korea; Division of Pediatrics and Perinatology, Faculty of Medicine, Tottori University, Tottori 683-8504, Japan; Division of Therapeutic Development for Intractable Bone Diseases, Graduate School of Medicine and Faculty of Medicine, The University of Tokyo, Tokyo 113-0033, Japan; Department of Pediatrics, Graduate School of Medicine, Osaka University, Osaka 565-0871, Japan; Department of Medical Statistics, Graduate School of Medicine, Osaka Metropolitan University, Osaka 545-8585, Japan; Department of Medical Statistics, Graduate School of Medicine, Osaka Metropolitan University, Osaka 545-8585, Japan; Medical Affairs Department, Kyowa Kirin Co., Ltd., Tokyo 100-0004, Japan; Medical Affairs Department, Kyowa Kirin Co., Ltd., Tokyo 100-0004, Japan; Department of Diabetes and Endocrinology, Tamaki-Aozora Hospital, Tokushima 779-3125, Japan; Center for Promoting Treatment of Intractable Diseases, ISEIKAI International General Hospital, Osaka 530-0052, Japan

**Keywords:** bone turnover markers, PTH/Vit D/FGF23, osteomalacia, rickets, disorders of calcium/phosphate metabolism

## Abstract

X-linked hypophosphatemia (XLH) is a genetic disease that results in excessive FGF23, chronic hypophosphatemia, and musculoskeletal abnormalities, with affected patients experiencing symptoms such as bone pain, bone deformity, fracture, and pseudofracture. Burosumab is a fully human monoclonal antibody that binds to FGF23, improving lowered serum 1,25(OH)_2_D and phosphate levels in patients with XLH. There are insufficient data on the use of burosumab, its safety, and the outcomes of treated patients in a real-world setting. The SUNFLOWER (Study of longitUdinal observatioN For patients with X-Linked hypOphosphatemic rickets/osteomalacia in collaboration With Asian partnERs) study is an ongoing longitudinal, observational cohort study of patients with XLH in Japan and South Korea. Enrollment occurred between April 2018 and December 2020. This interim analysis compared the background characteristics of patients who received burosumab with those who did not, and assessed improvements in biomarkers, physical and motor function, health-related quality-of-life (HRQOL) and other patient-reported outcome (PRO) measures, as well as the safety of burosumab treatment in 143 Japanese patients from 15 institutions over 6 mo. The patients had a median [interquartile range] age of 17.5 [11.0, 38.8] yr and 98 (68.5%) were female. Among patients aged <18 and ≥18 yr, 40/73 (54.8%) and 25/70 (35.7%) received burosumab, respectively. More patients aged ≥18 who received burosumab had bone pain at baseline vs those not treated with burosumab (6/25, 24.0% vs 2/45, 4.4%, *p*=.021). Patients treated with burosumab had improved serum phosphate and 1,25(OH)_2_D levels; moreover, rickets severity and HRQOL/PRO measures, such as pain, appeared to improve over 6 mo of burosumab treatment, and no new safety concerns were identified. This study identified trends in the background characteristics of patients with XLH who receive burosumab in real-world clinical practice. Furthermore, the results support the use of burosumab therapy in real-world settings.

## Introduction

X-linked hypophosphatemia (XLH) is a serious debilitating genetic disease that is characterized by musculoskeletal abnormalities. The disease is rare, with an incidence of approximately 1 in 20 000 to 60 000.^1-3^ It is caused by loss-of-function mutations in the phosphate-regulating gene with homologies to endopeptidases on the X chromosome (*PHEX*), which lead to excessive FGF23 levels, chronic hypophosphatemia, and bone mineralization disorders.^4^^,^^5^ Hypophosphatemia and insufficient bone mineralization may contribute to the varied symptoms experienced by patients with XLH, including dental abscesses, rickets, and abnormal gait in children, and enthesopathy, bone pain, and osteomalacia in adults.^4^^,^^5^ Patients with XLH are often treated with active vitamin D and/or oral phosphate. However, these conventional therapies do not persistently address the elevated FGF23 levels and thus do not restore normal serum phosphate levels in patients.^4^^,^^6^ The long-term use of these treatments can lead to secondary hyperparathyroidism, tertiary hyperparathyroidism, and nephrocalcinosis.^4^ Some patients require corrective surgeries for limb deformities and may undergo spinal decompression and joint replacements.^7-10^

Burosumab is a fully human monoclonal antibody that binds to FGF23, thereby increasing renal reabsorption of phosphate, elevating serum 1,25(OH)_2_D levels, and increasing serum phosphate levels.^11^ Previous clinical trials have shown that treatment with burosumab improves phosphate homeostasis in adults and children with XLH, thus enhancing measures such as Rickets Severity Score (RSS), stiffness, and healing of fractures/pseudofractures.^12-15^ Burosumab has been approved in several countries since 2018 for the treatment of XLH.^16^ Approval in South Korea was granted in September 2020, and the drug was launched in South Korea in May 2023 for pediatric XLH patients. In Japan, burosumab was approved in December 2019 for the treatment of FGF23-related hypophosphatemic rickets and osteomalacia and is expected to improve symptoms in affected patients.^16^^,^^17^ However, the course and burden of the disease are not fully understood. Additionally, there are insufficient data on the pattern of burosumab prescription, its safety, and the outcomes of treated patients in a real-world setting.

The SUNFLOWER (Study of longitUdinal observatioN For patients with X-Linked hypOphosphatemic rickets/osteomalacia in collaboration With Asian partnERs) study is an ongoing longitudinal, observational cohort study of patients with XLH in Japan and South Korea.^18^ This interim analysis of the SUNFLOWER study examined the background characteristics of Japanese patients who received burosumab and those who did not. Furthermore, this analysis assessed any improvement in biomarkers, physical and motor function, health-related quality-of-life (HRQOL) and patient-reported outcome (PRO) measures, as well as the safety of burosumab treatment in Japanese patients over 6 mo.

## Materials and methods

### Study design and treatment

The detailed SUNFLOWER study protocol has been previously reported.^18^ Briefly, the SUNFLOWER study is a longitudinal, observational cohort study of patients with XLH in Japan and South Korea; the observational period was from the time of enrollment (between April 01, 2018 and April 30, 2022) until April 30, 2028, for a maximum of 10 yr. For this interim analysis, data between April 01, 2018 and December 31, 2020 from patients enrolled at 15 medical institutions in Japan were analyzed. As this was an observational study, patients received treatment for XLH at their physician’s discretion and depending on the treatment options and regimens available in their country. The SUNFLOWER study is being conducted in accordance with the ethical principles that have their origin in the Declaration of Helsinki, and all relevant national regulations in Japan (including the Ethical Guidelines for Medical and Health Research Involving Human Subjects) and South Korea. Patients were informed about the purpose of the study, and written informed consent was obtained; additional written consent was obtained from the parents or legal guardians of patients aged <20 yr. This study was registered with the identifiers NCT03745521 and UMIN000031605.

### Patients

The full patient inclusion and exclusion criteria have been previously reported.^18^ Eligible patients were of any age; had a clinical diagnosis of XLH with a documented *PHEX* mutation, or a first-degree relative with a documented *PHEX* mutation, or a confirmed intact FGF23 level > 30 pg/mL; and had current or previous physical examination findings or laboratory and radiographical findings of rickets or osteomalacia (regardless of treatment history). Patients who were participating in another clinical study at the time of informed consent were excluded. To obtain a wide spectrum of general XLH data, there were no selection or exclusion criteria based on the specific stage or severity of the disease.

### Study assessments

The background information of patients who did and did not receive burosumab treatment were compared; as a result, this analysis included only Japanese participants, as burosumab had not been launched in South Korea at the time of this analysis. For patients who received burosumab, background characteristics were defined as data from immediately prior to burosumab initiation; for those who did not receive burosumab, background characteristics were defined as data from the start of the study. The dose of burosumab was assessed following 3 and 6 mo of treatment in patients who received burosumab. The burosumab dose was determined based on the last dose given in 3 time periods: baseline (the date of burosumab treatment initiation after informed consent); 3 mo (from day 1 to day 90); and 6 mo (from day 91 to day 240). As the dose was only recorded when the dose changed, visits with no recorded dose were considered to have the same dose as the previous visit. Additionally, the number of adverse events and complications related to XLH with up to 6 mo of burosumab treatment were recorded. Disease-related complications included new complications that occurred during the first 6 mo after initiating burosumab treatment. Random blood and urine sampling was performed at least 4 h after a meal or intake of a phosphate preparation in principle. Laboratory measurements taken at baseline, 3 mo, and 6 mo included serum and urine phosphate, serum 1,25(OH)_2_D, serum 25(OH)D, albumin, serum and urine calcium, serum intact PTH (iPTH), creatinine, and intact FGF23 levels (Determiner CL FGF23, Minaris Medical). Additionally, serum alkaline phosphatase (ALP) levels were measured in patients aged <18 yr, and serum bone-specific ALP (BALP) levels were measured in patients aged ≥18 yr. The change in motor function was assessed at baseline and at 6 mo with the 6-min walk test (patients aged <18 yr) and the Timed Up & Go (TUG) test (patients aged ≥18 yr). The severity of rickets for patients aged <18 yr was measured using the RSS assessment^19^^,^^20^ at baseline, 3 mo, and 6 mo. HRQOL and PROs for patients aged <18 yr were assessed at baseline and at 6 mo using the Revised Faces Pain Scale (FPS-R),^21^ the 10-item Short Form (SF-10) health survey Physical Summary Scale (PHS^22^; standardized using US norms^23^), and the Palliative Performance Scale (PPS).^24^ Patients aged ≥18 yr were assessed at baseline and at 6 mo using the Brief Pain Inventory (BPI)^25^ and Western Ontario and McMaster Universities Osteoarthritis Index (WOMAC) subscale scores for pain, stiffness, and physical function.^26^^,^^27^ The WOMAC scores were adjusted to a potential maximum of 100 using methods described in the user guide.^27^

### Statistical analyses

Patients were categorized into groups according to the presence or absence of burosumab treatment, and the distributions of characteristics were compared between the groups. For patients who received burosumab, baseline characteristics were determined from the data from the date of treatment initiation, and for patients who did not receive burosumab, data from the date of informed consent were used. Categorical variables were analyzed as the number of patients and corresponding percentages. Pearson’s chi-square test was used to compare distributions between groups, and Fisher’s exact test was used in instances where the expected frequency of any variable was <5. Continuous variables were summarized using medians and interquartile ranges, and comparisons between groups were made with the Mann–Whitney U-test.

Comparisons were conducted between the subpopulations by age (<18 yr and ≥18 yr) at baseline. As this interim analysis was conducted to describe the observed safety over a short period of time, *p*-values for efficacy outcomes were not calculated and no formal statistical comparisons between groups were conducted. Furthermore, the incidence of the new disease-related complications in patients who received burosumab was described with frequencies and percentages. Changes of motor functions and indicators of HRQOL and PRO at each follow-up period were compared with mean and standard deviation. Changes in biomarkers for phosphate metabolism and bone turnover, as well as RSS over 6 mo were described with mean and standard deviation. All statistical analyses were conducted using R (version 4.3.0) statistical software.^28^

## Results

### Patients

The patients’ backgrounds and characteristics are tabulated in Table 1. Among the 143 patients in this interim analysis, 65 (45.5%) were treated with burosumab and 78 (54.5%) received treatment other than burosumab (including active vitamin D and/or oral phosphates). Of the patients treated with burosumab, the majority (63/65, 96.9%) had received prior treatment for XLH. Most patients who did not receive burosumab continued active vitamin D and/or oral phosphate therapy (74/78, 94.9%). Less than half of the patients aged ≥18 yr were treated with burosumab (25/70, 35.7%), and approximately half of the patients aged <18 yr were treated with burosumab (40/73, 54.8%). Among patients aged ≥18 yr with bone pain at baseline, more patients were treated with burosumab than not (6/25, 24.0% vs 2/45, 4.4%, *p* = .021). Additionally, among patients aged ≥18 yr with hyperparathyroidism and calcification (including hypercalciuria, kidney stones, and nephrocalcinosis) at baseline, a higher percentage was treated with burosumab than those who were not (hyperparathyroidism: 14/25, 56.0% vs 7/45, 15.6%, *p*<.001; calcification: 16/25, 64.0% vs 12/45, 26.7%, *p* = .002). Patients aged <18 yr who were treated with burosumab had higher median (interquartile range) serum ALP levels (1397 [1024, 1787] vs 865 [552, 1572] U/L, *p* = .019) and higher RSS (2.50 [0.88, 3.53] vs 1.19 [0.88, 1.78], *p* = .13) at baseline than those who were not treated with burosumab. Patients aged ≥18 yr who were treated with burosumab had lower median (interquartile range) phosphate levels (2.10 [1.90, 2.30] vs 2.40 [2.10, 2.70] mg/dL, *p* = .003), higher scores on the BPI (worst pain in last 24 h: 4.00 [2.00, 6.00] vs 1.00 [0.00, 3.00], *p* = .008), and longer time on the TUG test (10.65 [9.45, 14.91] vs 9.84 [9.09, 10.93] s, *p* = .043) at baseline, than those not receiving burosumab.

**Table 1:** Patient backgrounds and baseline characteristics.

	**Total** **(*N* = 143)**	**Burosumab-treated** **(*n* = 65)**	**No burosumab treatment** **(*n* = 78)**	***p*-value** ^**a**^
Age, yr	17.5 [11.0, 38.8]	15.0 [7.5, 36.0]	18.8 [13.6, 41.4]	.035
< 18 yr^b^	73 (51.0)	40 (61.5)	33 (42.3)	.022
Sex, female	98 (68.5)	43 (66.2)	55 (70.5)	.6
*PHEX* mutation	70 (49.0)	29 (44.6)	41 (52.6)	.3
Family history of *PHEX* mutation	38 (26.6)	19 (29.2)	19 (24.4)	.5
**< 18 yr, *n***	73	40	33	
Age, yr	11.0 [6.0, 14.5]	9.0 [5.4, 14.0]	13.0 [7.5, 15.0]	.089
Height, cm	134.3 [104.6, 147.1]	122.5 [96.7, 143.8]	141.0 [117.3, 149.4]	.025
Height, Z-score	−1.91 [-2.40, -1.24]	−2.12 [−2.56, −1.52]	−1.62 [−2.28, −1.01]	.073
BW, kg	33.4 [18.9, 46.8]	25.6 [17.2, 44.3]	41.6 [26.5, 48.0]	.046
BW, Z-score	−0.34 [−1.00, 0.28]	−0.47 [−1.17, 0.18]	−0.26 [−0.68, 0.37]	.1
Phosphate, mg/dL	2.60 [2.20, 3.20]	2.50 [2.18, 2.93]	3.00 [2.25, 3.35]	.2
Serum 1,25(OH)_2_D, pg/mL	50.40 [37.85, 67.60]	46.60 [35.08, 56.95]	55.50 [49.20, 69.45]	.018
Serum 25(OH)D, ng/mL	17.0 [14.5, 21.2]	15.5 [13.8, 18.3]	21.0 [16.5, 24.0]	.002
Albumin, g/dL	4.60 [4.50, 4.90]	4.70 [4.60, 4.90]	4.60 [4.45, 4.70]	.038
Calcium, mg/dL	9.50 [9.30, 9.70]	9.50 [9.30, 9.70]	9.50 [9.15, 9.75]	.8
Serum ALP, U/L	1286 [832, 1684]	1397 [1024, 1787]	865 [552, 1572]	.019
Serum iPTH, pg/mL	46.0 [33.5, 63.0]	45.5 [33.0, 62.3]	52.0 [38.0, 65.0]	.4
Creatinine, mg/dL	0.38 [0.30, 0.49]	0.36 [0.25, 0.43]	0.48 [0.37, 0.56]	.002
Intact FGF23, pg/mL	149.00 [106.00, 239.50]	140.00 [102.95, 208.25]	163.00 [136.00, 269.00]	.2
RSS	1.69 [0.88, 2.88]	2.50 [0.88, 3.53]	1.19 [0.88, 1.78]	.13
6-min walk test, m	451.50 [390.00, 504.25]	453.00 [389.50, 496.50]	450.00 [394.00, 528.00]	.7
Abnormal gait	33 (45.2)	22 (55.0)	11 (33.3)	.064
Bone deformation	41 (56.2)	22 (55.0)	19 (57.6)	.8
Bone pain	6 (8.2)	5 (12.5)	1 (3.0)	.064
Calcification^c^	23 (31.5)	9 (22.5)	14 (42.4)	.068
Ectopic ossification	16 (21.9)	10 (25.0)	6 (18.2)	.5
Hearing impairment	0 (0.0)	0 (0.0)	0 (0.0)	
History of bone fractures	5 (6.8)	2 (5.0)	3 (9.1)	.7
Hyperparathyroidism	4 (5.5)	2 (5.0)	2 (6.1)	>.9
Hypertension	0 (0.0)	0 (0.0)	0 (0.0)	
Renal dysfunction	0 (0.0)	0 (0.0)	0 (0.0)	
Surgical history^d^	17 (23.3)	9 (22.5)	8 (24.2)	.9
**≥ 18 yr, *n***	70	25	45	
Age, yr	39.3 [24.6, 48.1]	42.0 [30.0, 50.5]	37.5 [20.5, 45.5]	.14
Height, cm	151.5 [145.1, 157.6]	148.6 [144.1, 156.7]	152.1 [145.4, 158.6]	.6
Height, Z-score	−2.18 [−2.89, −1.23]	−2.31 [−3.55, −1.70]	−2.13 [−2.74, −0.96]	.2
BW, kg	52.5 [45.5, 58.3]	54.6 [49.3, 60.3]	51.0 [44.9, 54.6]	.071
BW, Z-score	−0.72 [−1.28, 0.20]	0.09 [−1.06, 0.43]	−0.89 [−1.40, 0.00]	.075
Phosphate, mg/dL	2.30 [2.00, 2.53]	2.10 [1.90, 2.30]	2.40 [2.10, 2.70]	.003
Serum 1,25(OH)_2_D, pg/mL	46.1 [36.3, 55.3]	44.1 [33.2, 53.2]	46.8 [37.8, 58.0]	.3
Serum 25(OH)D, ng/mL	15.5 [13.0, 20.0]	15.0 [12.0, 17.0]	16.0 [14.0, 21.5]	.13
Albumin, g/dL	8.70 [4.60, 27.30]	8.40 [4.70, 16.40]	8.75 [3.75, 33.08]	.7
Calcium, mg/dL	7.00 [3.75, 12.10]	7.00 [3.90, 11.90]	7.30 [3.70, 14.15]	.7
Serum iPTH, pg/mL	67.5 [48.8, 96.0]	81.0 [64.0, 109.0]	57.0 [48.0, 86.0]	.057
Creatinine, mg/dL	0.55 [0.47, 0.68]	0.54 [0.48, 0.65]	0.55 [0.47, 0.71]	.6
Intact FGF23, pg/mL	150.50 [96.25, 326.50]	170.00 [135.00, 278.00]	134.00 [85.75, 401.50]	.4
TUG test, s	10.31 [9.22, 12.41]	10.65 [9.45, 14.91]	9.84 [9.09, 10.93]	.043
BPI worst pain	3.00 [0.00, 5.00]	4.00 [2.00, 6.00]	1.00 [0.00, 3.00]	.008
Abnormal gait	9 (12.9)	6 (24.0)	3 (6.7)	.06
Bone deformation	14 (20.0)	7 (28.0)	7 (15.6)	<.001
Bone pain	8 (11.4)	6 (24.0)	2 (4.4)	.021
Calcification^c^	28 (40.0)	16 (64.0)	12 (26.7)	.002
Ectopic ossification	30 (42.9)	17 (68.0)	13 (28.9)	.002
Hearing impairment	1 (1.4)	1 (4.0)	0 (0.0)	.4
History of bone fractures	22 (31.4)	10 (40.0)	12 (26.7)	.2
Hyperparathyroidism	21 (30.0)	14 (56.0)	7 (15.6)	<.001
Hypertension	13 (18.6)	6 (24.0)	7 (15.6)	.5
Renal dysfunction	7 (10.0)	2 (8.0)	5 (11.1)	>.9
Surgical history^d^	26 (37.1)	13 (52.0)	13 (28.9)	.055

Data are median [interquartile range] or *n* (%), unless otherwise stated, and include measurements taken more than 90 d prior to the date of burosumab treatment initiation (burosumab-treated) or date of obtaining informed consent (no burosumab treatment).

a Pearson’s chi-square test/Fisher’s exact tests were used for categorical variables, whereas the Mann–Whitney U-test was used for continuous variables.

b Age at either time of obtaining informed consent or initiation of burosumab treatment.

c Including hypercalciuria, kidney stones, and nephrocalcinosis.

d Surgeries related to X-linked hypophosphatemia.

Abbreviations: ALP, alkaline phosphatase; BPI, Brief Pain Inventory; BW, body weight; iPTH, intact PTH; *PHEX,* phosphate-regulating gene with homologies to endopeptidases on the X chromosome; RSS, Rickets Severity Score; TUG, Timed Up & Go.

The burosumab dose categories are shown in Table 2. The percentage of patients aged <18 yr who received a higher dose of burosumab increased over time, with 10.0% (4/40), 37.1% (13/35), and 41.4% (12/29) receiving >1.0 mg/kg at baseline, 3 mo, and 6 mo, respectively.

**Table 2:** Burosumab dose.

	**< 18 yr (Q2W)**			**≥ 18 yr (Q4W)**		
	**Baseline** **(*n* = 40)**	**3** mo **(*n* = 35)**	**6** mo **(*n* = 29)**	**Baseline** **(*n* = 25)**	**3** mo **(*n* = 22)**	**6** mo **(*n* = 20)**
Burosumab dose, mg/kg, median [interquartile range]	0.81 [0.73, 0.92]	0.90 [0.76, 1.14]	0.93 [0.81, 1.13]	0.97 [0.93, 1.07]	0.99 [0.94, 1.07]	0.99 [0.94, 1.08]
Burosumab dose category, *n* (%)						
< 0.6 mg/kg	3 (7.5)	3 (8.6)	2 (6.9)			
0.6–< 1.0 mg/kg	33 (82.5)	19 (54.3)	15 (51.7)			
≥ 1.0 mg/kg	4 (10.0)	13 (37.1)	12 (41.4)			
< 0.8 mg/kg				3 (12.0)	2 (9.1)	1 (5.0)
0.8–< 1.2 mg/kg				21 (84.0)	20 (90.9)	17 (85.0)
≥ 1.2 mg/kg				1 (4.0)	0 (0.0)	2 (10.0)

Abbreviations: Q2W, once every 2 wk; Q4W, once every 4 wk.

### Safety outcomes in the burosumab-treated group

The incidence of disease-related complications following burosumab treatment is shown in Table 3. Five new disease-related complications were reported by 6 mo, of which 4 were ectopic ossifications and 1 was a case of hyperparathyroidism. Three serious adverse events had occurred by the 6-mo assessment (lumbago, intermittent exotropia [monocular], and acute cholecystitis); all of these resolved with treatment during the observational period.

**Table 3:** Complications over 6 mo of burosumab treatment (*n* = 65).

	**Baseline**	**3** mo	**6** mo	**New cases at** **6** mo
Ectopic ossification^a^	27 (41.5)	27 (41.5)	28 (43.1)	4 (6.2)
Bone deformation	29 (44.6)	29 (44.6)	29 (44.6)	0 (0.0)
Calcification^b^	25 (38.5)	25 (38.5)	25 (38.5)	0 (0.0)
Abnormal gait	28 (43.1)	28 (43.1)	28 (43.1)	0 (0.0)
Hyperparathyroidism	16 (24.6)	17 (26.2)	17 (26.2)	1 (1.5)
Bone pain	11 (16.9)	11 (16.9)	11 (16.9)	0 (0.0)
Hypertension	6 (9.2)	6 (9.2)	6 (9.2)	0 (0.0)
Renal dysfunction	2 (3.1)	2 (3.1)	2 (3.1)	0 (0.0)
Hearing impairment	1 (1.5)	1 (1.5)	0 (0.0)	0 (0.0)

Data are *n* (%).

a Including enthesopathy, ligament ossification, osteoarthritis, joint pain, and joint stiffness.

b Including hypercalciuria, kidney stones, and nephrocalcinosis.

### Biomarker and motor function assessments in the burosumab-treated group

**Figure 1:**
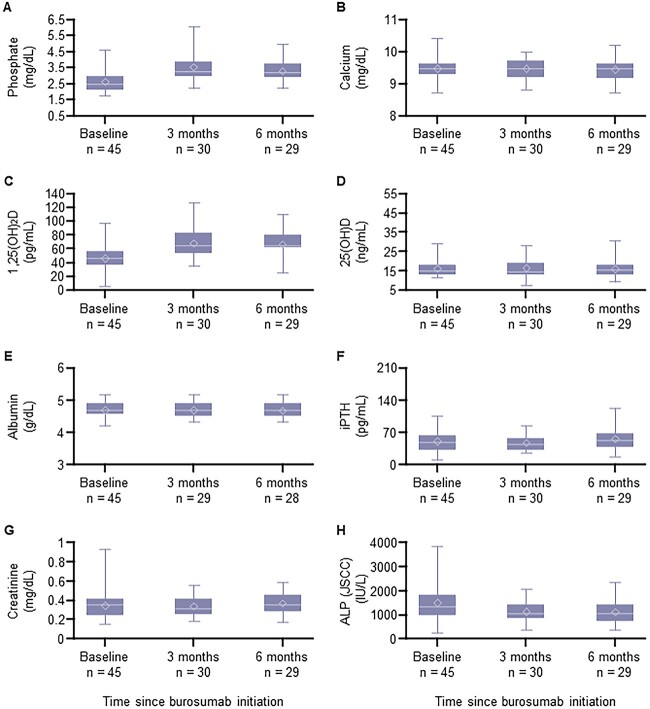
Changes in biomarkers for phosphate metabolism and bone turnover over 6 mo of burosumab treatment in patients aged <18 yr. (A) Phosphate levels. (B) Calcium levels. (C) 1,25(OH)_2_D levels. (D) 25(OH)D levels. (E) Albumin levels. (F) iPTH levels. (G) Creatinine levels. (H) ALP levels. The horizontal line indicates the median, upper and lower box edges show the interquartile range, whiskers represent minimum and maximum values, and the diamond represents the mean. ALP levels were determined using the JSCC method. ALP, alkaline phosphatase; iPTH, intact PTH; JSCC, Japan Society of Clinical Chemistry.

**Figure 2:**
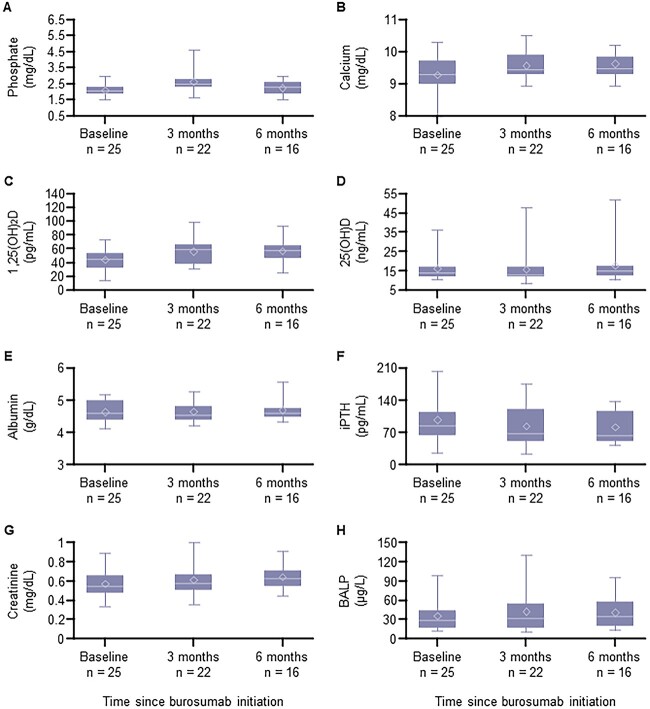
Changes in biomarkers for phosphate metabolism and bone turnover over 6 mo of burosumab treatment in patients aged ≥18 yr. (A) Phosphate levels. (B) Calcium levels. (C) 1,25(OH)_2_D levels. (D) 25(OH)D levels. (E) Albumin levels. (F) iPTH levels. (G) Creatinine levels. (H) BALP levels. The horizontal line indicates the median, upper and lower box edges show the interquartile range, whiskers represent minimum and maximum values, and the diamond represents the mean. BALP, bone-specific alkaline phosphatase; iPTH, intact PTH.

**Figure 3:**
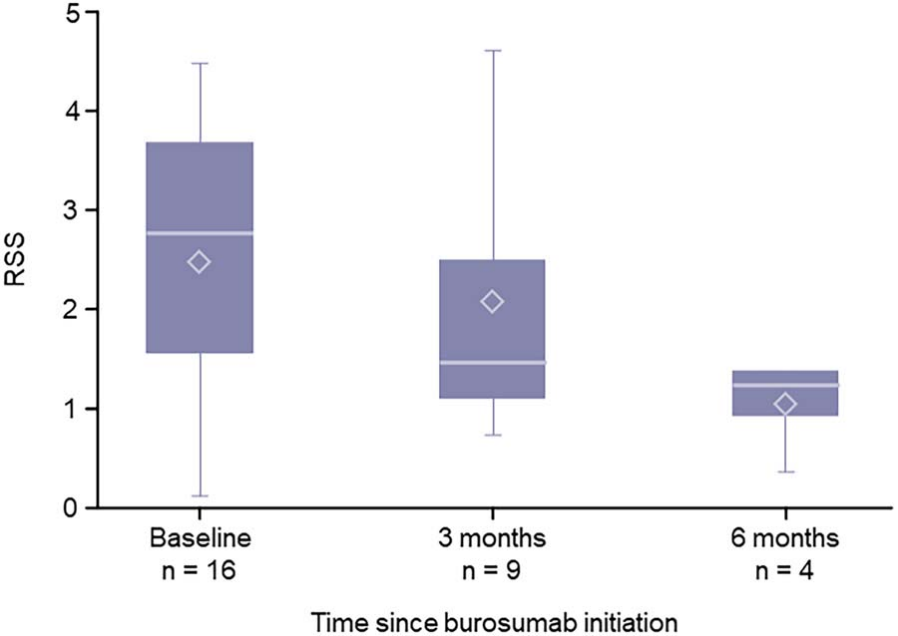
Changes in RSS over 6 mo of burosumab treatment in patients aged <18 yr. The horizontal line indicates the median, upper and lower box edges show the interquartile range, whiskers represent minimum and maximum values, and the diamond represents the mean. RSS, Rickets Severity Score.

The biomarker measures for patients under and over 18 yr old are shown in Figures 1 and 2, respectively. Serum phosphate and 1,25(OH)_2_D levels tended to increase following burosumab treatment in both age groups. Serum ALP levels in patients aged <18 yr tended to decrease following burosumab treatment (Figure 1H), while serum BALP levels in patients aged ≥18 yr remained consistent throughout 6 mo of burosumab treatment (Figure 2H). There were no changes in the 6-min walk test (patients aged <18 yr) and the TUG test (patients aged ≥18 yr) following 6 mo of burosumab treatment (Table 4). The severity of rickets tended to decrease over time with burosumab treatment (Figure 3).

**Table 4:** Change in motor function of patients with X-linked hypophosphatemic rickets/osteomalacia after 6 mo of burosumab treatment.

	**< 18 yr (Q2W)**		**≥ 18 yr (Q4W)**	
	**Baseline** ^a^ **(*n* = 34)**	**6** mo **(*n* = 21)**	**Baseline** ^a^ **(*n* = 23)**	**6** mo **(*n* = 16)**
6-min walk test, m	445.9 ± 75.9	421.9 ± 109.1		
TUG test, s			12.9 ± 6.7	11.8 ± 5.0

Data are mean ± SD. The 6-min walk test was conducted in patients <18 yr, and the TUG test was conducted in patients ≥18 yr.

a Including measurements from up to 90 d prior to baseline.

Abbreviations: Q2W, once every 2 wk; Q4W, once every 4 wk; TUG, Timed Up & Go.

### HRQOL and PRO assessments in the burosumab-treated group

The HRQOL and PRO assessments at baseline and 6 mo after burosumab treatment are shown in Table 5. The BPI worst pain and average pain scores in patients aged ≥18 yr were numerically lower following 6 mo of burosumab treatment. The WOMAC subscale scores for pain, stiffness, and physical function also tended to improve after burosumab treatment. The FPS-R, SF-10 PHS, and PPS scores for patients aged <18 yr were similar at baseline and after 6 mo of burosumab treatment.

**Table 5:** Change in QOL and PRO of patients with X-linked hypophosphatemic rickets/osteomalacia after 6 mo of burosumab treatment.

	**< 18 yr (Q2W)**		**≥ 18 yr (Q4W)**	
	**Baseline** ^**a**^ **(*n* = 35)**	**6** mo **(*n* = 21)**	**Baseline** ^**a**^ **(*n* = 25)**	**6** mo **(*n* = 16)**
FPS-R score	1.5 ± 1.1	1.3 ± 0.6		
SF-10 PHS score	47.8 ± 7.6	47.0 ± 7.4		
SF-10 PPS score	53.2 ± 5.5	53.1 ± 6.4		
BPI worst pain			4.2 ± 2.5	2.8 ± 2.6
BPI average pain			2.8 ± 2.1	1.8 ± 1.9
WOMAC subscale score, pain			35.7 ± 22.5	19.1 ± 19.3
WOMAC subscale score, stiffness			44.0 ± 31.8	24.6 ± 34.0
WOMAC subscale score, physical function			28.7 ± 21.3	17.2 ± 19.5

Data are mean ± SD. The FPS-R and SF-10 were used to evaluate patients <18 yr, and the BPI and WOMAC were used to evaluate those ≥18 yr. The WOMAC subscale values were adjusted to a potential maximum combined score of 100.

a Including measurements from up to 90 d prior to baseline.

Abbreviations: BPI, Brief Pain Inventory; FPS-R, Revised Faces Pain Scale; PHS, Physical Summary Scale; PPS, Palliative Performance Scale; PRO, Patient-Reported Outcome; QOL, Quality-of-life; Q2W, once every 2 wk; Q4W, once every 4 wk; SF-10, 10-item Short Form health survey; WOMAC, Western Ontario and McMaster Universities Osteoarthritis Index.

## Discussion

This interim analysis of the SUNFLOWER study found that patients who received burosumab tended to have biomarker measurements consistent with more severe disease and had more complications at baseline, including gait abnormalities and bone pain. Patients who were treated with burosumab showed initial improvements in serum phosphate and 1,25(OH)_2_D levels; moreover, rickets severity and HRQOL/PRO measures, such as pain, appeared to improve over 6 mo of burosumab treatment. Among the patients who received burosumab, the percentage who developed new disease-related complications was low. Although some patients experienced serious adverse events after burosumab treatment, all resolved during the observational period, and none resulted in death. Thus, our findings suggest that burosumab in patients with XLH has an acceptable safety profile in a real-world setting.

A higher percentage of younger patients (aged <18 yr) received burosumab than adults (54.8% vs 35.7%), suggesting that younger patients may be more likely to receive treatment with burosumab. Moreover, pediatric patients who were treated with burosumab had a higher ALP level at baseline than those who did not receive burosumab. This may indicate a difference in the severity of rickets, although higher ALP levels may also reflect the younger age of patients treated with burosumab.^29^ A higher frequency of adult patients with hyperparathyroidism and calcification (including hypercalciuria, kidney stones, and nephrocalcinosis), which can occur in patients with XLH including those treated with oral phosphate and active vitamin D therapy,^4^ was present in the burosumab-treated group at baseline than in the non-burosumab group. This suggests that physicians seem to choose to initiate burosumab treatment in patients with greater disease severity or XLH-related complications.

All treatments in this study were provided at the site at the physician’s discretion, including any native vitamin D supplementation prior to the start of the study. In Japan, the use of native vitamin D supplements to treat vitamin D deficiency is uncommon because native vitamin D supplements are not available via prescription. Therefore, we believe that only some of the patients in this study received native vitamin D supplementation. At baseline, the median serum 25(OH)D level was low (15.5 ng/mL), further indicating that supplementation was not often used.

Although conventional therapy can provide symptomatic improvements in XLH, the effects may be insufficient, and some patients will still experience symptoms such as bone pain, bone deformity, fracture, and pseudofracture.^30-32^ In this interim analysis, we observed that patients who received burosumab treatment for 6 mo had increased serum phosphate and 1,25(OH)_2_D levels and decreased ALP levels, which is consistent with previous clinical trials of burosumab.^12^^,^^33-35^ Additionally, the rickets severity of patients aged <18 yr and HRQOL and pain measures of those aged ≥18 yr tended to improve following burosumab treatment, in line with previous reports.^12^^,^^33^^,^^34^ The findings from this interim analysis provide evidence that improvement in biomarker measurements occurs rapidly following burosumab treatment in a real-world setting. However, we did not identify any motor function improvements. As patients received other therapies prior to initiating burosumab treatment, the 6-mo duration may have been too short to achieve further motor function improvements. Further longer term studies are expected to reveal whether there are any additional improvements with burosumab treatment beyond 6 mo.

We acknowledge the limitations of this study. Missing data are an unavoidable problem in observational studies; therefore, the number of patients differed at some timepoints. In addition, this analysis included patients with a short follow-up period who started burosumab after enrollment. Thus, the timing of burosumab initiation varied among patients. Although all eligible patients had a diagnosis of XLH, some included patients had intact FGF23 levels >30 pg/mL, but did not have confirmed *PHEX* mutations in either themselves or a first-degree relative. As this interim analysis only included patients who had received burosumab for 6 mo, the effectiveness and safety of burosumab in a real-world practice beyond 6 mo remain to be determined. Although the SUNFLOWER study included patients from both Japan and South Korea, this interim analysis was only performed on data from Japanese patients. Thus, our findings may not apply to patients in other countries.

In conclusion, the findings from this interim analysis of the longitudinal SUNFLOWER study suggested that burosumab tends to be administered to patients with greater disease severity, including bone pain or gait disturbance. Our data also provide evidence for the safety of burosumab for up to 6 mo and suggest an improvement in XLH symptoms in patients treated with burosumab in real-world clinical practice.

## Data Availability

Research data, including participant data, the statistical analysis plan, and informed consent forms, are not shared. The study protocol has been previously published.^18^

## Clinical Trial Registration

NCT03745521; UMIN000031605.
